# An innovative high-throughput genome releaser for rapid and efficient PCR screening

**DOI:** 10.3389/fbioe.2025.1547909

**Published:** 2025-03-25

**Authors:** Guoliang Yuan, Aljon Salalila, Sungjoo Hwang, Zhiqun Daniel Deng, Shuang Deng

**Affiliations:** ^1^ Energy and Environment Directorate, Pacific Northwest National Laboratory, Richland, WA, United States; ^2^ Department of Naval Architecture and Marine Engineering, University of Michigan, Ann Arbor, MI, United States

**Keywords:** Squash-PCR, high-throughput, *Aspergillus niger*, 96-well plate, automated workflows, DNA extraction

## Abstract

High-throughput PCR screening is vital in synthetic biology and metabolic engineering, enabling rapid and precise analysis of genetic modifications. However, current methods face challenges including inefficient DNA extraction, high variability across sample types, scalability limitations, and the high cost of template DNA extraction. To address these common challenges, we developed a High-Throughput Genome Releaser (HTGR). This innovative device utilizes a squash-based method for rapid, cost-effective, and efficient DNA extraction, optimized for subsequent PCR reactions. After testing various synthetic materials, we selected a plastic that closely mimics the smooth surface and compression properties of microscope slides, ensuring reliable and consistent performance. The device comprises a 96-well plate and a Shear Applicator, designed for both manual and automated operation, and is compatible with standard liquid-handling robotic platforms. This compatibility simplifies integration into high-throughput PCR workflows. Additionally, we developed software to support its automated functions. Our results demonstrated that the specially engineered 96-well plate and HTGR effectively squash fungal spores, releasing sufficient genomic DNA for PCR screening with 100% efficiency. The genome releaser enables the preparation of PCR-ready genomic DNA from 96 samples within minutes, eliminating the need for an extraction buffer. Its adaptability to a wide range of microorganisms and cell types makes it a versatile tool that could significantly advance biomanufacturing processes.

## 1 Introduction

Advances in synthetic biology and biomanufacturing enable the rapid engineering of metabolic pathways and the introduction of new biosynthetic pathways, unlocking biological routes to the production of both known and novel compounds. These innovations frequently generate hundreds to thousands of mutant strains, necessitating fast and efficient genotype screening to ensure the accuracy of strain construction. PCR remains the gold standard for verifying DNA edits. Conventional PCR screening relies on genomic DNA, and extracting DNA from fungal sources is labor-intensive, time-consuming, and requires the use of toxic chemicals (Leslie and Summerell, 2008; Rodrigues et al., 2018).

**FIGURE 1:**
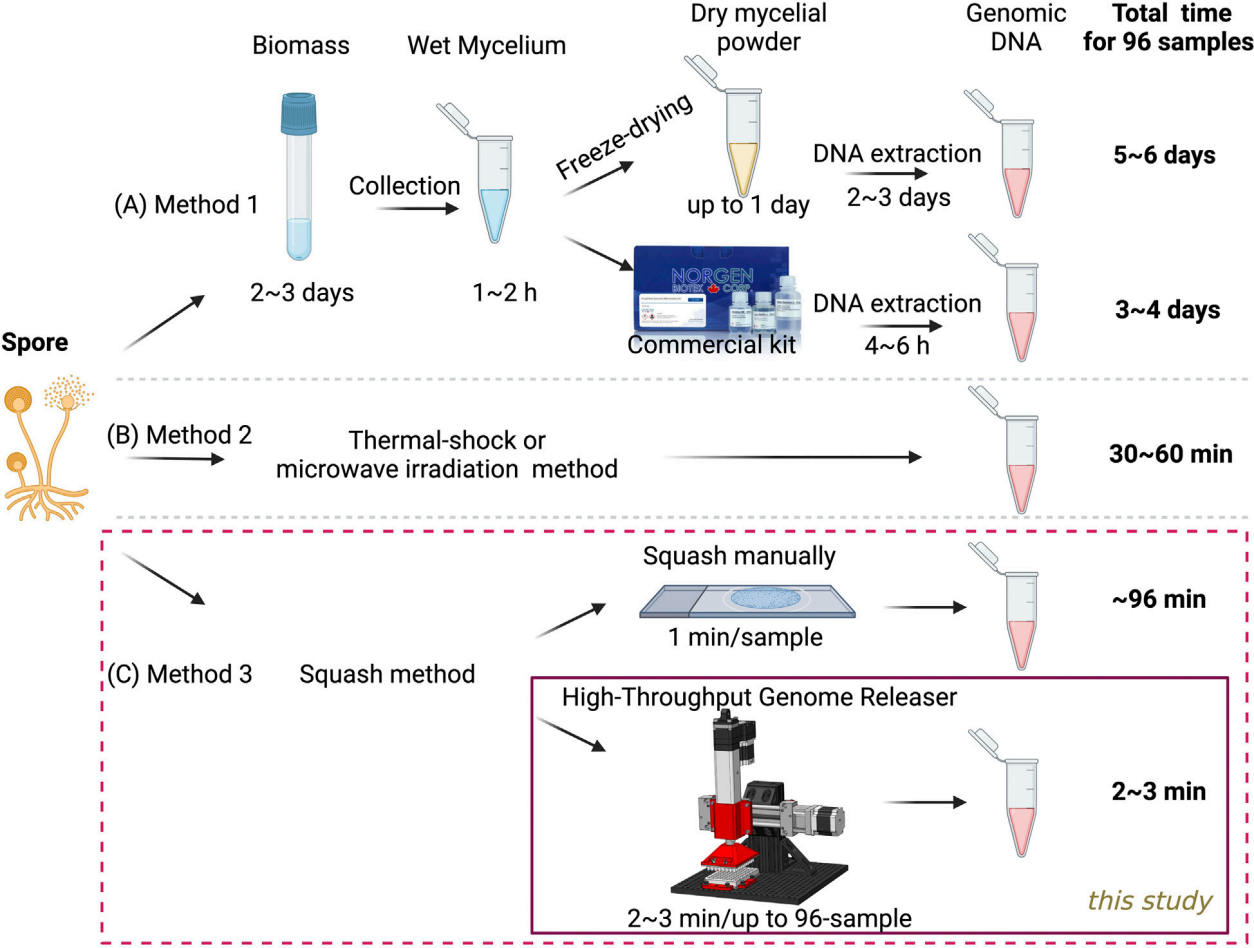
Comparison of various methods for preparing 96 genomic DNA templates from fungal spores. **(A)** Traditional method used for fungal genomic DNA extraction from biomass. **(B)** Published method for genomic DNA release directly from spores and mycelium **(C)** A novel high-throughput method developed in this study for genomic DNA release directly from spores.

Fungal cell walls are rigid, composed of chitin and glucan (Garcia-Rubio et al., 2019), and present a significant barrier to DNA extraction. Traditional methods usually require freezing in liquid nitrogen, grinding, or bead beating, combined with strong buffers such as Cetyltrimethylammonium Bromide (CTAB) to break the cell walls, followed by multiple steps for DNA extraction (Kumar and Mugunthan, 2018; Conlon et al., 2022; Dong et al., 2016; Haugland et al., 1999). Although some commercial kits have been developed to simplify the extraction process, DNA extraction from fungal sources still takes considerable time, often up to 5–6 days. This is particularly true when large numbers of strains need to be analyzed, as 2–3 days of culturing are required to produce sufficient fungal biomass (Figure 1A). While this lengthy method typically yields high-quality genomic DNA, it is not suitable for rapid PCR screening of large mutant samples. In contrast, numerous methods have been reported to significantly simplify the extraction process and reduce extraction time (Figure 1B). These approaches typically utilize thermal shock, microwave irradiation, or sonication in a water bath to disrupt fungal spores or mycelium (Jeon et al., 2023; Pilo et al., 2022; Fraczek et al., 2019; Ferreira et al., 1996). Each technique aims to effectively break down the cell walls, facilitating easier access to the cellular contents for subsequent PCR analysis, with processing times ideally reduced to 30–60 min. However, these methods are often limited to one or a few specific fungal strains or sample types, making them unlikely to be applied consistently across various fungal strains. When we implemented the thermal shock approach with *Aspergillus*, we observed significant variability in PCR results, highlighting the challenges of using these techniques for diverse fungal species. This inconsistency may stem from variations in cell wall composition and structure among different strains, necessitating a universal, efficient extraction method.

Previously, we developed a highly efficient slide-squashing method to disrupt spore cell walls, allowing the release of genomic DNA in about 1 minute per sample, without the need for any lysis or extraction buffer (Figure 1C, top panel). This technique has proven to be both rapid and highly effective, demonstrating broad applicability across various cell types, including fungi, yeast, and microalgae (Yuan et al., 2024a; Yuan et al., 2023). However, the scalability of the slide-squashing technique was limited by its manual nature. To address this limitation, we aim to develop in this study a genomic DNA releaser that facilitates rapid, cost-effective, and efficient DNA extraction, specifically optimized for high-throughput PCR workflows (Figure 1C, bottom panel). This system is designed to streamline the extraction process for high-throughput applications, ensuring consistent and sufficient DNA yields across a diverse array of fungal and microbial strains.

## 2 Results

### 2.1 Design of 1-well and 96-well plates for manual sample squashing

**FIGURE 2:**
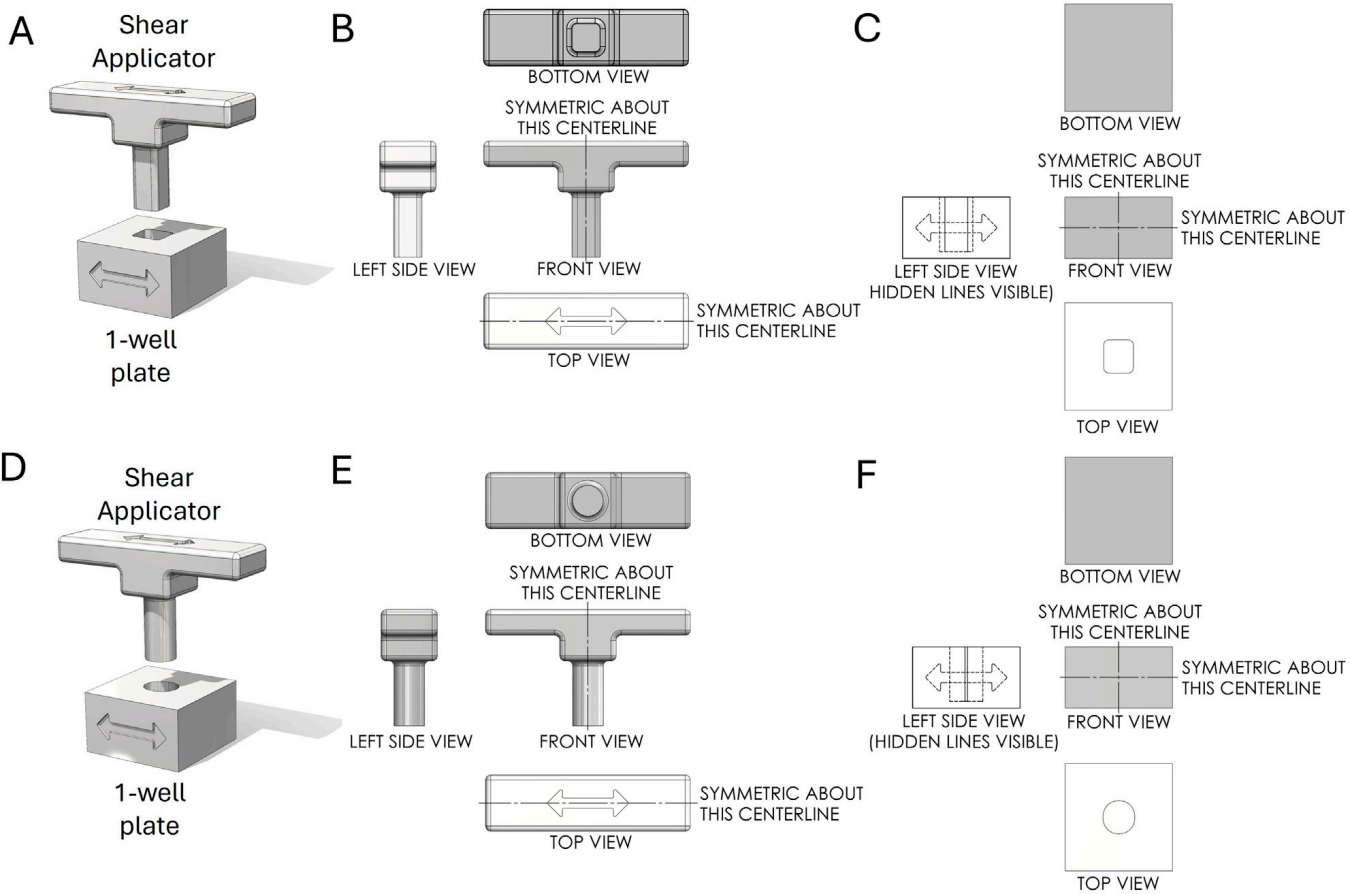
First-angle projection of the Shear Applicator pin and 1-well plate. **(A)** Assembly of the Shear Applicator with a rectangular pin and square bottom, positioned over the 1-well plate. **(B)** Technical drawing of the Shear Applicator. **(C)** Technical drawing of the 1-well plate. **(D)** Assembly of the Shear Applicator with a cylindrical pin and flat bottom, positioned over the 1-well plate. **(E)** Technical drawing of the Shear Applicator. **(F)** Technical drawing of the 1-well plate.

The primary objective in designing the 1-well plate was to replicate the squashing motion used in the glass slide and cover slip method developed by Yuan et al., (2024a), Yuan et al., (2023), where the 1-well plate serves as the glass slide and the Shear Applicator pin serves as the cover slip. Various shapes were explored, each aimed at optimizing contact area, pressure distribution, and mechanical disruption of the sample during the squashing process. The different designs considered were:

1. Rectangular Pin with Square Bottom: This design aimed to maximize the contact area with the sample, ensuring uniform pressure distribution during the squashing process (Figures 2A–C).
2. Cylindrical Pin with Flat Bottom: This design featured a cylindrical shape for easy insertion into the well, providing a straightforward geometry for consistent manufacturing and testing (Figures 2D–F).
3. Cylindrical Pin with Flat Bottom and Filleted Edges: Similar to the previous design but with filleted edges to reduce stress concentrations and improve the durability of the pin.
4. Cylindrical Pin with Spherical Bottom: This shape was intended to concentrate the applied force at a single point initially, spreading out as the pin was pressed down, potentially improving the disruption of tougher samples.
5. Cylindrical Pin with Diamond Bottom: This design featured a diamond-shaped bottom to introduce additional shear forces during the squashing process, aiming to enhance the mechanical disruption of the sample.

**FIGURE 3:**
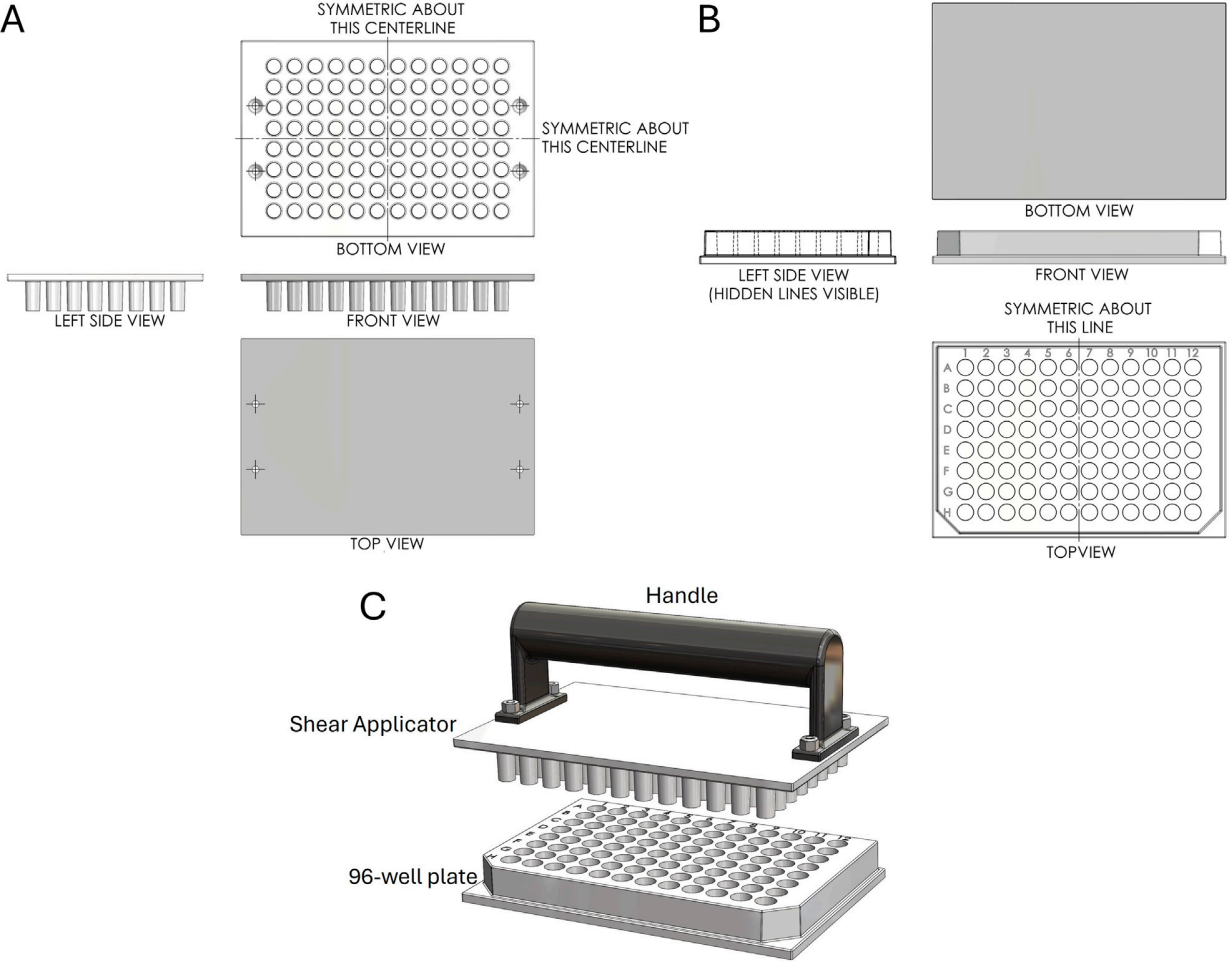
First-angle projection of the Shear Applicator and 1-well plate. **(A)** Technical drawing of the Shear Applicator with 96 pins. **(B)** Technical drawing of the 96-well plate. **(C)** Assembly view of the Shear Applicator with a handle, positioned over the 96-well plate.

Next, we designed a 96-well plate along with a corresponding Shear Applicator featuring a cylindrical pin with a flat bottom (Figure 3A). This pin design was chosen based on the most favorable outcomes from testing, as outlined in the following section. It aligns with the standard 96-well plate format, ensuring compatibility with traditional plate designs while enabling effective sample processing. Unlike typical 96-well plates, which are often shelled to minimize material use, this design features a solid bottom, enhancing the plate’s rigidity to withstand significant vertical and lateral forces during the mechanical squashing of samples to break cells (Figure 3B). The Shear Applicator is equipped with an integrated handle for easy operation, and the handle was 3D-printed in-house using Polylactic Acid (PLA) to balance lightweight construction with sufficient strength for manual use (Figure 3C). Both the 96-well plate and the Shear Applicator were designed to integrate with our High-Throughput Genome Releaser (HTGR) machine, supporting both manual and automated sample processing workflows. The dimensions conform to standard microplate size specifications, ensuring compatibility with common laboratory equipment such as robotic arms or automated pipetting systems. In addition, the 96-well plate has been optimized for mass production through injection molding. The walls feature a 1-degree inward draft angle, which facilitates easy removal from the mold during manufacturing. This draft is critical for preventing the walls from sticking to or shearing against the mold, ensuring consistent quality in high-volume production runs. These design considerations allow for efficient manufacturing while preserving the mechanical properties required for high-force applications in both manual and automated sample squashing.

### 2.2 Implementation and evaluation of 1-well plate for spore squashing-driven Squash-PCR

**FIGURE 4:**
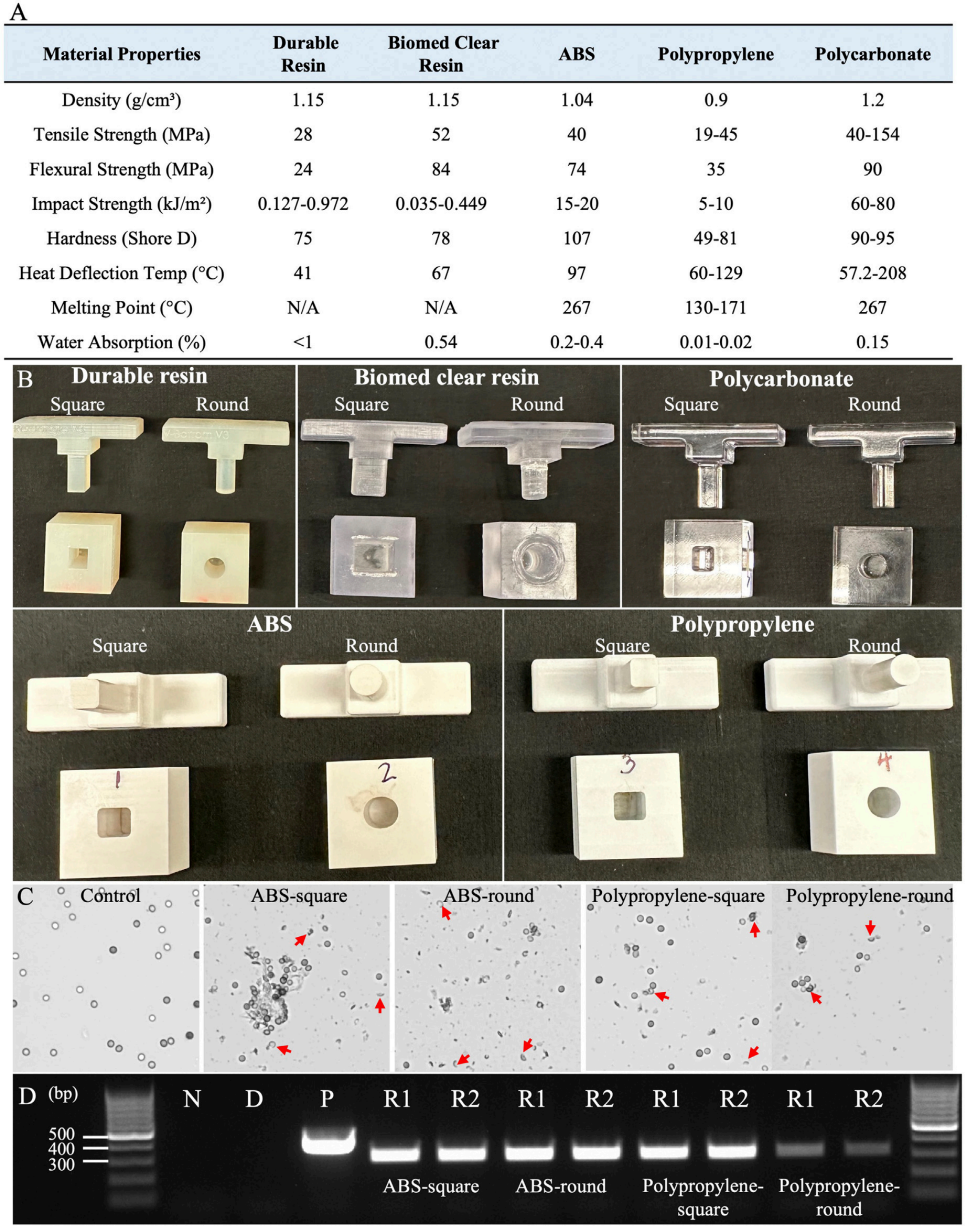
Materials screening and 1-well plate testing for effective Squash-PCR. **(A)** The material properties of five different substances were evaluated for suitability in the squashing mold (Xometry, 2024a; Xometry, 2024b; Xometry, 2024c; Formlabs, 2020a; Formlabs, 2020b). **(B)** Five different materials were manufactured in two shapes—square and round—to examine their efficacy in spore squashing. **(C)** Microscopic analysis of spore squashing using ABS and polypropylene wells. The spores that were not subjected to squashing served as the control. Red arrows indicate the broken spores after squashing. **(D)** PCR analysis of spore squashing was conducted using the 123_F and 124_R primers for the *oahA* mutant. Lane N: negative control (water); Lane D: direct PCR with unsquashed spores; Lane P is positive control (wild-type genomic DNA); Lanes R1 and R2: replicate 1 and replicate 2, respectively.

This slide-based approach enables efficient cell disruption and rapid genomic DNA extraction from fungal spores, owing to the slide’s ultra-smooth surface and high hardness (Yuan et al., 2023). To develop a 96-well plate designed for high-throughput genome release across various organisms, our first step involved identifying a material with the hardness and flatness of a microscope slide, as manufacturing a glass 96-well plate presents technical and economic challenges. The 1-well plate designs were initially fabricated using PLA for ease of 3D printing and rapid prototyping, featuring five different shapes described above. Each Shear Applicator pin was precisely matched to the internal geometry of the 1-well plate to ensure a secure fit for consistent shear stress application. Initial testing on tough spore samples, such as *Aspergillus niger*, aimed to evaluate the device’s ability to break open spores and release genomic DNA suitable for PCR testing. It was found that the Rectangular Pin with a Square Bottom and the Cylindrical Pin with a Flat Bottom enabled smoother motion compared to the other designs. However, the rough surface finish inherent to PLA prototypes introduced irregularities in sample processing and hindered the smooth side-to-side movement of the Shear Applicator, resulting in inconsistent shear stress and minimal spore breakage. To address these challenges, the design was refined by enlarging the base, adding an ergonomic handle, and elongating the well opening to allow smoother pin movement. We then evaluated five different materials—Durable Resin, Biomed Clear Resin, Acrylonitrile Butadiene Styrene (ABS), Polypropylene, and Polycarbonate—each with varying properties such as density, tensile strength, flexural strength, impact strength, hardness, heat deflection temperature, melting point, and water absorption (Figure 4A).

Prototypes were reprinted using Stereolithography (SLA) 3D printers with Durable Resin and Biomed Clear Resin, and Computer Numerical Control (CNC) machined from ABS, polycarbonate, and polypropylene, which provided improved surface finish and mechanical properties, with each well designed in two shapes: square and round (Figure 4B). These prototypes were tested by loading *Aspergillus niger* spores and performing squashing, as described in Method 4.2. The squashed spore samples were then evaluated under a microscope according to Method 4.4. The 1-well plate made from Biomed Clear Resin proved to be non-wear-resistant, breaking down into a white powder during squashing that contaminated the samples and prevented visual confirmation of successful DNA squashing, likely due to its low impact strength (0.449 kJ/m^2^) (Figure 4A). Furthermore, both Durable Resin and Polycarbonate failed to break open the spores effectively. The 3D-printed Durable Resin had a hard but relatively rough surface, which likely resulted in insufficient compression force to adequately flatten the spores. Even though polycarbonate exhibited higher mechanical properties, including a tensile strength 154 MPa, it did not produce good results, likely due to the difficulty of achieving a smooth surface finish. In contrast, both ABS and polypropylene proved to be effective materials for spore squashing. Their balanced mechanical properties, including tensile strength (40 MPa for ABS and 45 MPa for polypropylene), flexural strength (74 MPa for ABS and 35 MPa for polypropylene), and impact resistance (20 kJ/m^2^ for ABS and 10 kJ/m^2^ for polypropylene), enabled them to effectively break open the spores and release DNA for PCR testing. After spore squashing, both showed a significantly higher rate of spore breakage under the microscope compared to the control group, which had no broken spores. Notably, 1-well plates made from ABS produced more abundant spore debris than those made from Polypropylene, indicating enhanced spore disruption (Figure 4C). This visible debris highlights ABS’s superior performance in facilitating cell rupture compared to the other materials tested. Subsequently, Squash-PCR was performed to evaluate the effectiveness of spore squashing. ABS-square, ABS-round, and polypropylene-square 1-well plates all yielded bright PCR bands comparable to the positive control, indicating efficient genomic DNA release and successful amplification (Figure 4D). In contrast, the polypropylene-round 1-well plate produced weaker PCR bands, suggesting less effective spore disruption and DNA extraction (Figure 4D). These results indicate that both ABS, in round and square designs, and polypropylene, particularly in square-well designs, are suitable materials for developing a 96-well plate optimized for Squash-PCR applications. ABS’s thermal stability at 97°C and ability to achieve a fine surface finish made it ideal for consistent shear stress application. Additionally, its ease of machining, cost-effectiveness, and availability made ABS an ideal choice for prototype development and scaling up production.

### 2.3 96-well plate manual testing for Squash-PCR

**FIGURE 5:**
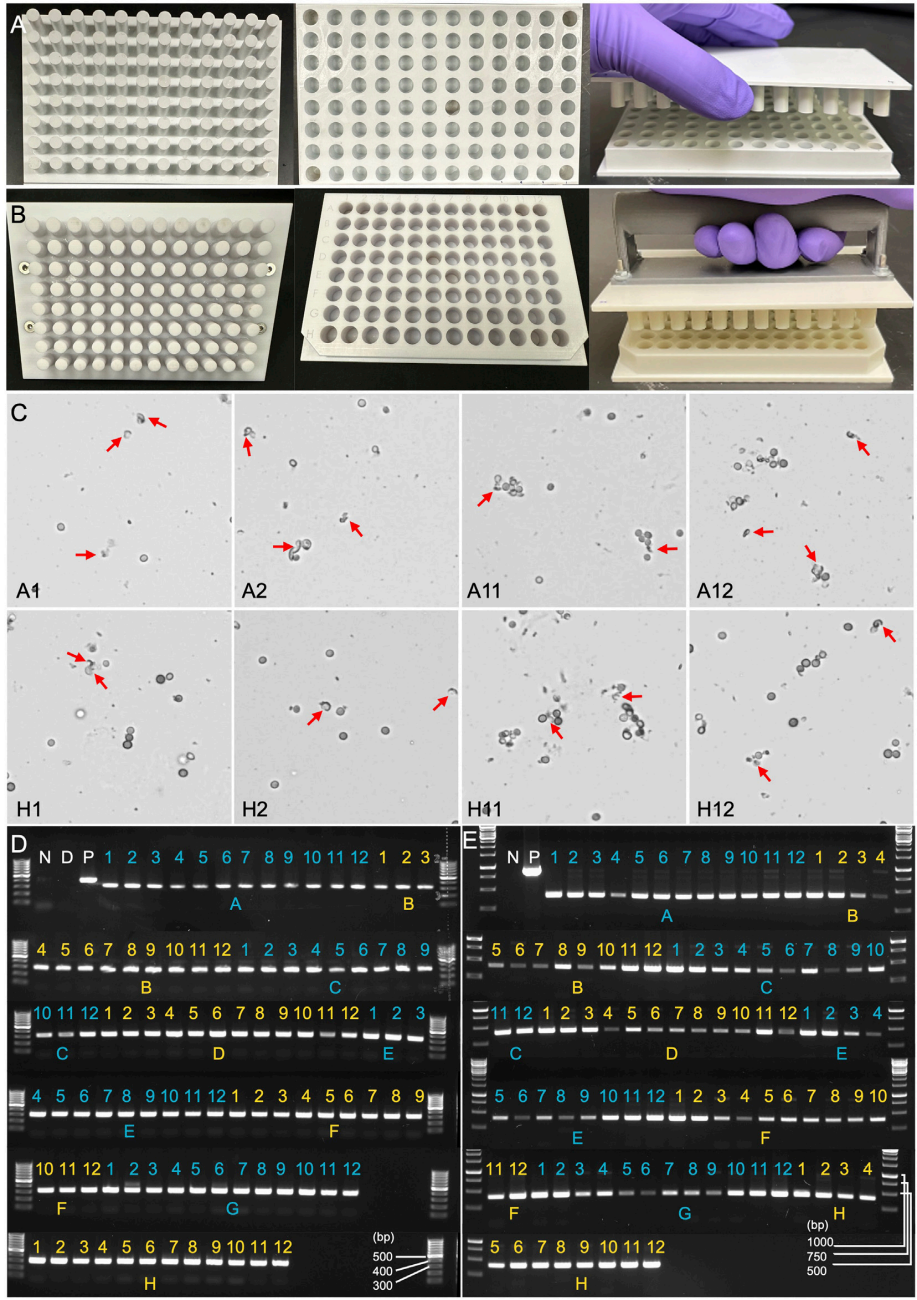
Evaluation of manually squashed fungal spores using microscopy and PCR genotyping. **(A)** Version 1.0 of the 96-well plate designed for Squash-PCR. The upper Plate contains 96 pins that align precisely with the 96-well plate. The bottom plate contains 96-well with excellent inner well flatness and whole plate flatness. **(B)** Version 2.0 of the 96-well plate for Squash-PCR with a standard microplate dimension. **(C)** Microscopic analysis of spores manually squashed using version 1.0 of the 96-well plate. Red arrows indicate the broken spores after squashing. A1, A2, A11, A12, H1, H2, H11, and H12 indicate the well positions on the 96-well plate. **(D)** PCR analysis of spores squashed manually using version 1.0 of the 96-well plate. The 123_F and 124_R primers were used for the genotyping of *oahA* mutant. N: negative control with water; D: direct PCR with un-squashed spores; and P: positive control using wild-type genomic DNA. **(E)** PCR analysis of spores squashed manually using version 2.0 of the 96-well plate. The PCR was conducted using the same protocol as described for panel D.

The 96-well plates with round wells are commonly used for various biological assays, including cell culture, PCR, and high-throughput screening applications. They are often preferred for their improved liquid handling, mixing, and pipetting accuracy. Therefore, we selected ABS material to manufacture a 96-well plate with round wells (version 1.0), specifically designed for Squash-PCR (Figure 5A). The machined components were manufactured with tolerances of ±0.15 mm and a surface roughness of 0.8 µm Ra. Following the same procedures established in 1-well plate testing, we added 5 µL of *Aspergillus niger* spores to each well and performed manual squashing as outlined in Method 4.3. After squashing, we evaluated the effectiveness of spore breakage using microscopy. For the initial test, we selected eight wells located in the four corners of the plate (A1, A2, A11, A12, H1, H2, H11, and H12) for squashing and microscopic assessment. Successful spore breakage was observed in all eight wells tested (Figure 5C), consistent with the spore breakage seen in the 1-well plate test (Figure 4C). To further assess the plates’ performance, we conducted Squash-PCR across all 96 wells of the Plate. Compared to the positive control, all 96 wells yielded bright PCR bands, indicating successful DNA amplification (Figure 5D). However, during testing, we observed that the distance between wells was larger than the standard 96-well plate format, rendering it (version 1.0) incompatible with standard electronic multichannel pipettes. To improve compatibility with multichannel pipettes, plate readers, and liquid handling robotics, we redesigned the plate and developed version 2.0 in a standardized format (Figure 5B). In this updated version, we also added a handle to the Shear Applicator to facilitate easier manual squashing and sliding. The new version (2.0) underwent testing with manual spore squashing, and the spore breakage observed was comparable to that seen with version 1.0. In addition to consistent spore breakage, further testing demonstrated that version 2.0 also produced high yields and reliable PCR results, achieving 100% efficiency across all wells (Figure 5E). This confirmed that the improvements in plate design, including the standardized well spacing and the handle on the Shear Applicator, streamlined the process while maintaining the effectiveness of both the spore squashing and downstream PCR amplification.

### 2.4 Cross-contamination assessment in Squash-PCR using the 96-well plate

**FIGURE 6:**
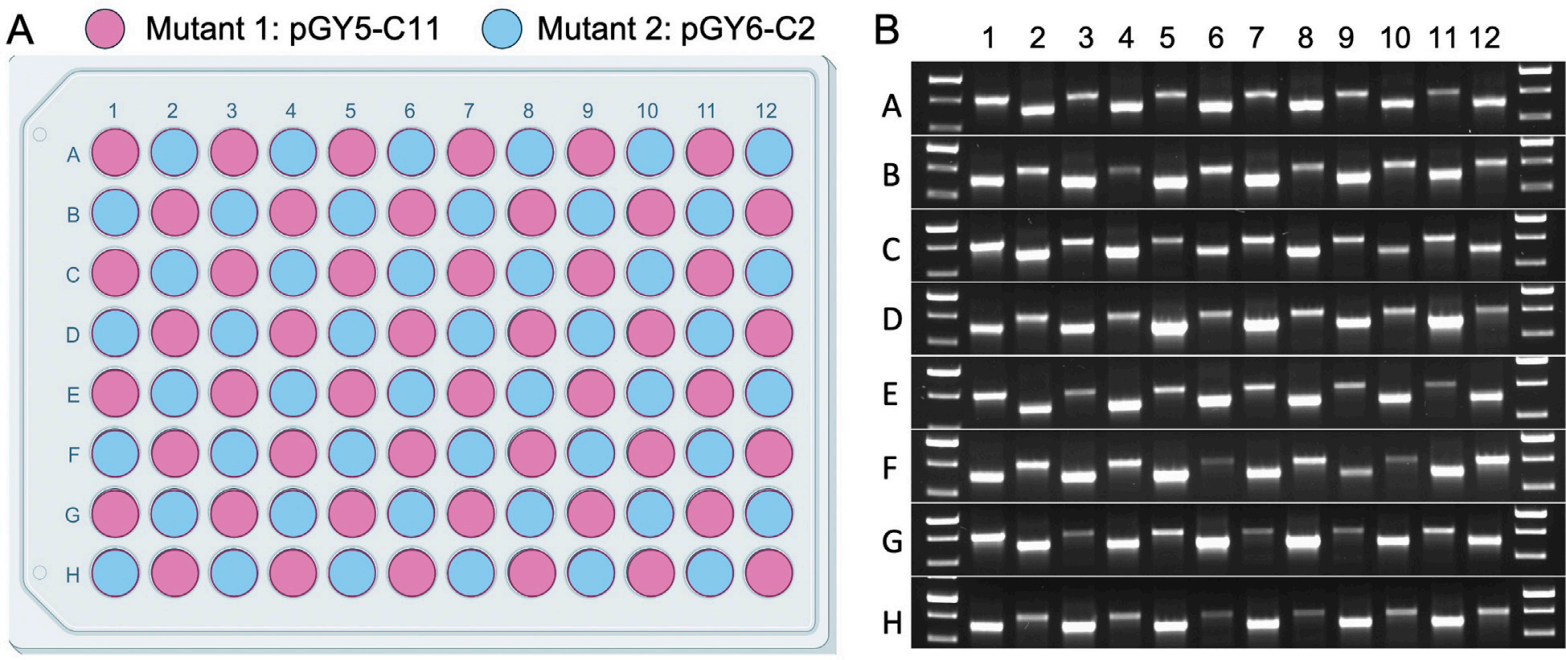
Cross-contamination assessment in Squash-PCR using the 96-well plate. **(A)** Plate layout of sample loading in the 96-well plate. The spores of 2 *A*. *niger albA* mutant (pGY5-C11 and pGY6-C2) were loaded into 96-well plate in a checkerboard layout. **(B)** Squash-PCR analysis of panel A with primers 11_F and 12_R. PCR bands of two different sizes were amplified due to differences in the deletion sizes resulting from gene editing.

Version 2.0 of the 96-well plate designed for spore squashing was thoroughly evaluated for the potential of cross-contamination during plate preparation. This evaluation involved loading the entire 96-well plate with spores from two distinct *albA* mutants, pGY5-C11 and pGY6-C2, followed by squashing the spores according to the protocol detailed in Method 4.3. A representative plate layout is shown in Figure 6A. To assess the effectiveness of the squashing process and detect any potential cross-contamination, the samples underwent Squash-PCR analysis. The PCR bands from the two mutants were expected to differ due to distinct deletion sizes unique to each strain. If cross-contamination had occurred, double bands would have been detected in individual wells. Following Squash-PCR, bright and distinct PCR bands were observed in all wells across the 96-well plate (Figure 6B), confirming the robustness and reliability of the Squash-PCR method. Notably, no double bands were detected in any of the wells (Figure 6B), indicating the absence of cross-contamination. These results demonstrate that version 2.0 of the 96-well plate effectively minimizes the risk of cross-contamination or carryover between wells, ensuring the integrity of the experimental process. The improved design not only facilitates precise and reliable sample processing but also maintains high standards of cleanliness and accuracy throughout high-throughput experiments.

### 2.5 Integrated hardware and software development for the high-throughput genome releaser system

**FIGURE 7:**
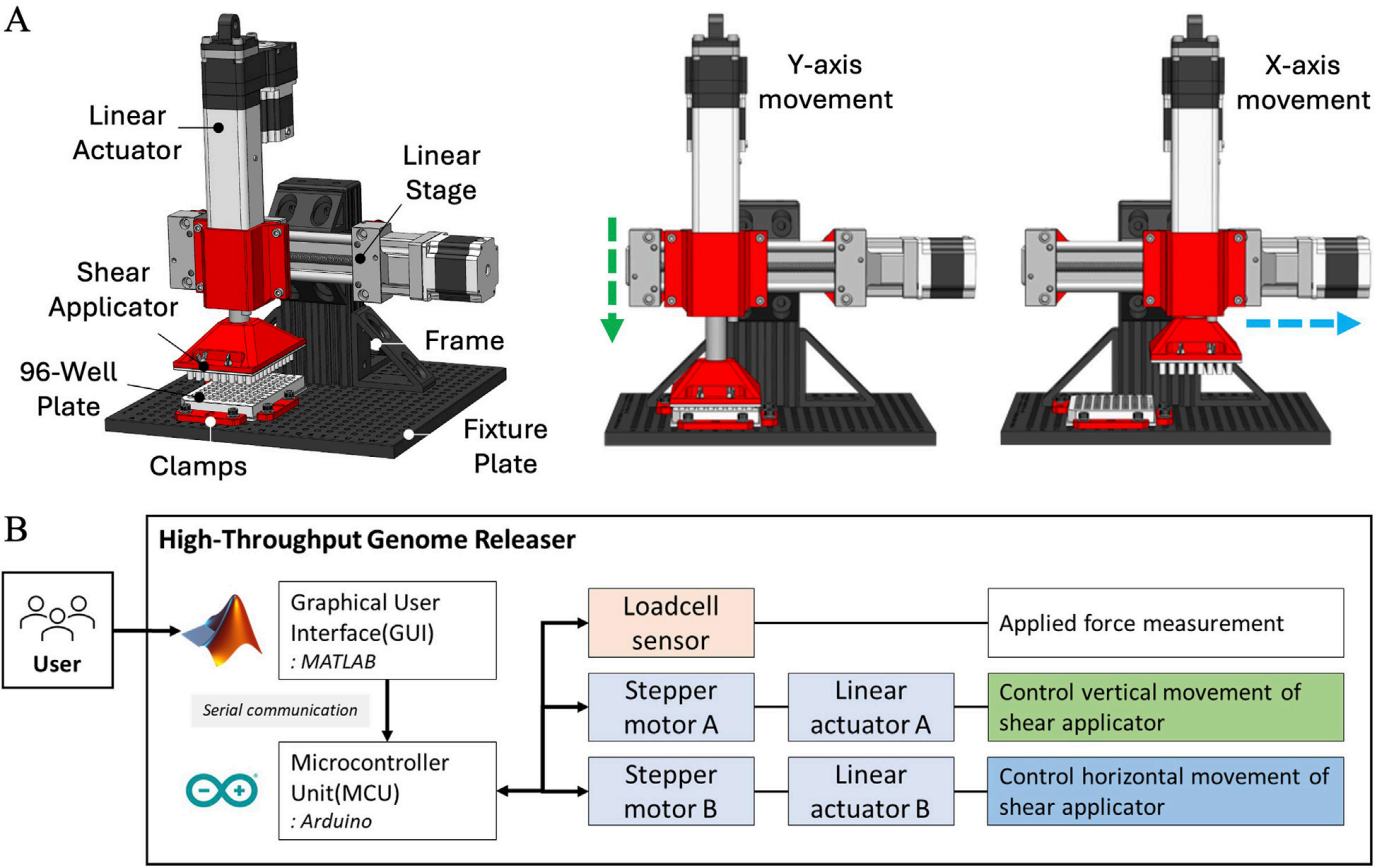
Schematic representation of HTGR and its hardware architecture. **(A)** Key components of the HTGR, including the linear actuator, linear stage, shear applicator, 96-well plate, clamps, fixture plate, and frame, along with controlled movements along the X and Y-axes for sample processing. **(B)** Hardware architecture of the HTGR, illustrating the system’s control setup, which includes the graphical user interface, microcontroller, load cell sensor, and stepper motors for controlling the Shear Applicator’s vertical and horizontal movements.

The HTGR system, comprising both the controller and software, is designed to automate the mechanical disruption of fungal spores for high-throughput applications. It mimics the Squash method developed by Yuan et al. (2024a), Yuan et al. (2023), leveraging precise mechanical actions to ensure consistent spore disruption for DNA extraction. The system measures the applied force and performs the mechanical disruption process (squashing) using stepper motors. On the hardware side, the HTGR system uses four Ω (OMEGA Engineering Inc., Norwalk, CT, United States) circular-type load cell sensors (LCMKD-200 N) and two pairs of stepper motors (NEMA 23, 173.5 in.-oz) equipped with linear actuators (NEMA 23, 100 mm stroke) supplied by McMaster-Carr (McMaster-Carr Supply Company, Elmhurst, IL, United States) (Figure 7A). The structural stability of the 8020-aluminum frame and the secure clamping of the 96-well plate ensure consistent mechanical action across all samples, preventing misalignment or variability in force application (Figure 7A). On the software side, MATLAB (MathWorks, 2024) App Designer is used for the graphical user interface (GUI), and the Arduino (Arduino, 2024) Mega R3 board is used for the microcontroller unit (MCU) (Figure 7B). The two interfaces communicate through serial communication.

The HTGR performs two primary functions: applying force and controlling the motion of the Shear Applicator. The controller moves the Shear Applicator horizontally and vertically, while the 96-well plate remains fixed (Figures 7A, B). A load cell sensor monitors the applied force and stops motion once the target force is reached. Once the Shear Applicator is aligned with the 96-well plate, it executes two programmed actions simultaneously—rubbing and stamping—to facilitate spore disruption. The rubbing motion moves the Shear Applicator horizontally while vertical force is applied, breaking spores within the wells. Meanwhile, the stamping motion periodically lifts and lowers the Shear Applicator, ensuring continuous force application while also redistributing liquid within the wells for uniform sample processing. This coordinated motion ensures consistent and effective spore disruption across all wells.

### 2.6 Evaluation of HTGR for Squash-PCR

**FIGURE 8:**
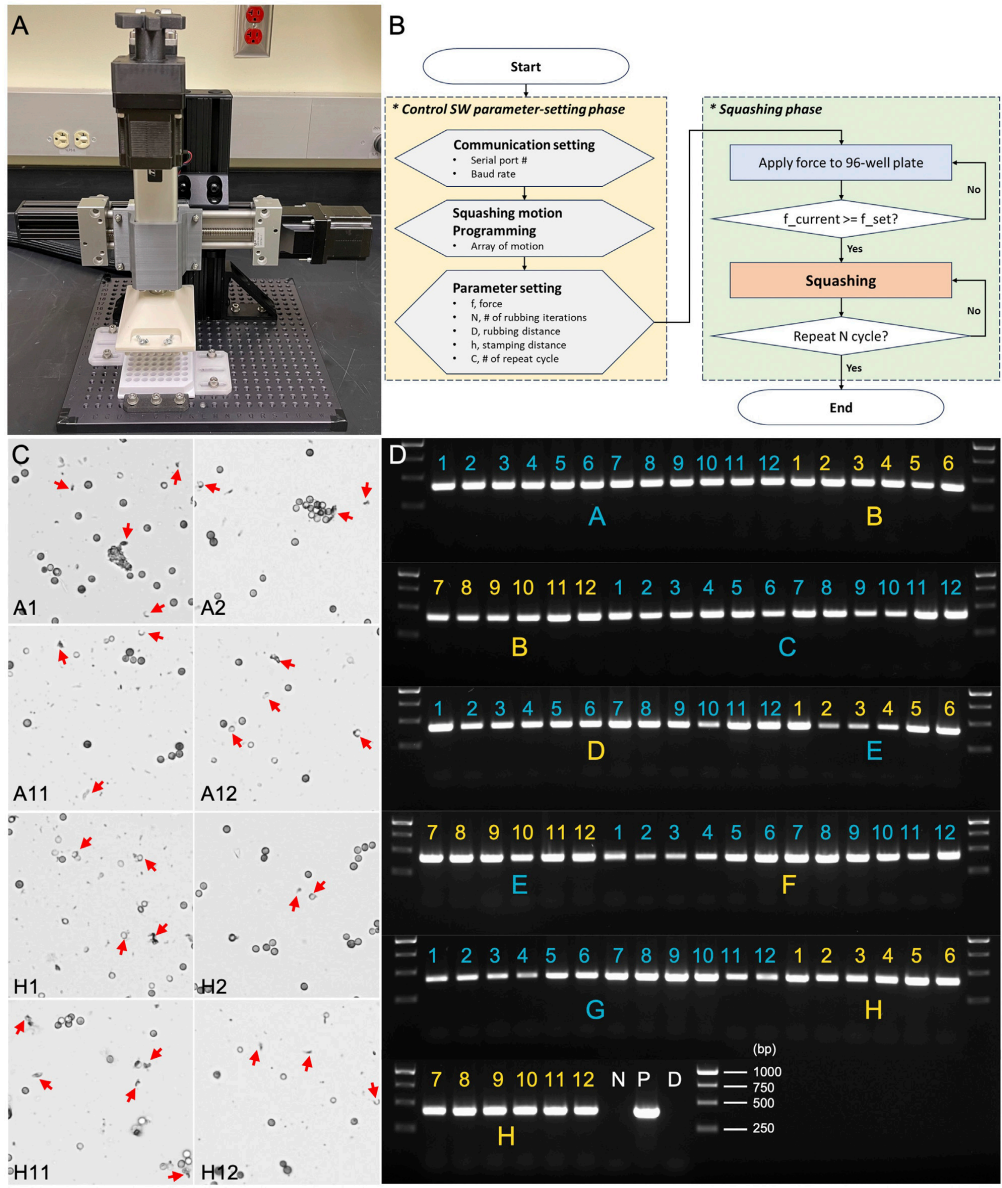
Assessment of HTGR using microscopy and PCR genotyping. **(A)** The automated HTGR system. **(B)** Flowchart of the HTGR software interface, illustrating the parameter-setting and squashing phases. **(C)** Microscopic analysis of spores squashed using version 2.0 of the 96-well plate and HTGR. Red arrows indicate the broken spores after squashing. **(D)** PCR analysis of spores squashed using version 2.0 96-well plate and HTGR. Primers 123_F and 124_R were used for the genotyping of the *oahA* mutant. N: negative control with water; D: direct PCR with un-squashed spores; and P: positive control using wild-type genomic DNA.

To enhance the efficiency and scalability of Squash-PCR, the automated HTGR system (Figure 8A) streamlines the manual squashing process, reducing hands-on time and ensuring consistency across experiments. The system applies precise mechanical force to squash fungal spores directly in 96-well plates, preparing them for downstream PCR analysis. Testing began with *Aspergillus niger* spores loaded into version 2.0 of the 96-well plate, following the workflow outlined by the HTGR software (Figure 8B). The HTGR user interface comprises two main phases: the Control SW parameter-setting phase and the Squashing phase. In the parameter-setting phase, users can adjust the force (F) and the number of interactions (N) to meet their specific experimental requirements. Once these parameters are defined and set, the user can initiate the process by clicking ‘Start.’ During the Squashing phase, the system automatically engages and performs the squashing operations based on the preset parameters. The HTGR is designed to enhance user convenience by automatically stopping once all the designated motions are completed, ensuring consistent and reliable squashing results without the need for continuous monitoring.

The spores were subjected to the automated squashing system, which was programmed to mimic the manual squashing protocol detailed in Method 4.3 but with added speed and precision. The entire plate was processed within minutes, which is comparable to the time typically required for manual squashing. Following a similar approach to our 96-well plate manual test, we selected eight wells positioned at the four corners of the plate (A1, A2, A11, A12, H1, H2, H11, and H12) for spore squashing and subsequent microscopic evaluation. In all tested wells, we observed remarkable spore breakage, demonstrating the effectiveness of the automated system (Figure 8C). We then expanded the testing to all 96 wells, conducting Squash-PCR after automated squashing to evaluate the quality of spore lysis and DNA release. PCR amplification was highly successful, with clear, bright bands observed in all wells across the 96-well plate (Figure 8D). The automated system maintained the same level of efficiency as the manual process, yielding consistent results and robust amplification across all wells tested. The application of a controlled vertical force of 294 N per well ensured uniform lysis of *Aspergillus niger* spores, providing reliable DNA extraction results in each sample. The combination of vertical squashing and lateral shearing forces further enhanced the mechanical disruption, leading to improved DNA release. The load cell feedback system maintained precise control over the applied forces, ensuring repeatability and accuracy in the extraction process. An additional benefit of the automated system was the reduced risk of contamination or variability due to human error, as the mechanical squashing process was standardized across all samples. No cross-contamination was detected, and PCR analysis confirmed that the system performed with 100% accuracy. Overall, the HTGR machine met the design goals, offering a scalable solution for high-throughput genomic analysis with reliable and reproducible DNA extraction, making it an ideal tool for large-scale studies where rapid and reliable sample processing is essential.

## 3 Discussion

The development of HTGR addresses a critical need for rapid, efficient, and scalable DNA extraction in synthetic and metabolic engineering, particularly for high-throughput PCR applications. As synthetic biology and bioengineering continue to grow in complexity and scale (McCarty and Ledesma-Amaro, 2019), the ability to analyze multiple constructs or microbial strains simultaneously is essential. However, current PCR screening techniques face several significant challenges. These include time-consuming DNA preparation, contamination risks, low consistency and reproducibility, and the need for automation to streamline large-scale sample collection and analysis. The HTGR’s innovative design, based on a squash method, provides a cost-effective and efficient solution to these hurdles, with versatile application potential across various microorganisms and cell types. The spore size and cell wall structure differences affect squashing efficiency. Rigid spores require more force for breakage, while delicate spores are more easily disrupted. The applied force depends on both the spores’ mechanical properties and the squashing plate material. Our design ensures consistent force distribution, but variations in spore size and toughness may impact efficiency. In manual squashing, the force can be easily adjusted. Future optimizations may involve increasing the maximum force of the HTGR and refining material properties, such as reducing surface roughness, to enhance compatibility with a broader range of microorganisms.

The effectiveness of the slide-based squashing method has been demonstrated in over 30 different strains of filamentous fungi, yeast, and microalgae, with *Aspergillus niger* being the most challenging species tested (Yuan et al., 2024a; Yuan et al., 2023). Our results validate the HTGR’s functionality in *Aspergillus niger* through a stepwise approach, starting with the design and fabrication of both 1-well and 96-well plates. Initial experiments focused on testing manual squashing with the 1-well plate, which enabled us to identify ABS material and flat-bottom cylindrical pins as optimal for constructing the 96-well plate, while polypropylene also demonstrated effectiveness for spore squashing. This groundwork was followed by the validation of the 96-well plate design for manual high-throughput applications. Both microscopy and 96-well PCR analysis confirmed that our ABS-based 96-well plate effectively disrupts *Aspergillus niger* spores, enabling highly efficient and reliable DNA extraction for PCR. The plate’s robust design ensures consistent spore lysis, yielding high-quality PCR products across multiple wells with minimal variability while eliminating the need for lysis and extraction buffers, thus significantly simplifying the workflow.

Cross-contamination is a significant concern when using 96-well plates, especially in high-sensitivity applications, as the close proximity of wells can lead to sample transfer between them (Walker, 2019). This risk is particularly elevated during sample preparation or extraction steps, where unintended transfer can potentially contaminate neighboring samples. A checkerboard pattern in 96-well plates is commonly employed in research to assess the risk of cross-contamination between wells under different experimental conditions (Forry et al., 2016; Bomsztyk et al., 2019). By utilizing a similar approach, we demonstrated that our 96-well plate effectively minimizes contamination risks. This capability is a significant advantage for preserving the integrity of high-throughput analyses, ensuring reliable results, and enhancing the reproducibility of experiments. To ensure cleanliness and prevent any residual debris from affecting subsequent samples, the plate is designed for single-use disposal, ensuring that each experiment is conducted with a fresh plate. This eliminates the risk of spore debris getting trapped in micro-scratches on the surface of pins or wells, which could otherwise lead to unintended carryover between different spore types. Using disposable plates also helps maintain consistency in squashing efficacy across experiments.

In addition to the hardware, the integration of specialized software for automating the HTGR was instrumental in enhancing its compatibility with standard robotic liquid-handling systems. This software enables automated operation, which not only increases throughput but also standardizes the squashing and DNA extraction process, improving reproducibility. The combined hardware and software system was tested with the automated High-Throughput Genome Releaser setup, which confirmed that the HTGR can perform Squash-PCR with 100% efficiency, processing 96 samples within minutes.

The HTGR presents a considerable advancement for high-throughput PCR workflows in biomanufacturing and microbial biology. Its compatibility with robotic systems makes it highly adaptable for industrial applications where processing large volumes of samples is required. By simplifying DNA extraction and providing a scalable, contamination-resistant design, the HTGR supports more efficient screening, enhances reproducibility, and reduces operational costs, all of which are essential for advancing research and development in synthetic biology and microbial engineering. For small-scale screenings, the 96-well plate can also be used manually, offering a lower-cost and more flexible option for researchers. Future studies could further explore the HTGR’s applicability to a broader range of cell types and its potential integration into fully automated pipelines for even greater throughput and precision. In addition to its high-throughput capabilities of PCR, the HTGR can potentially be readapted to handle larger sample volumes for DNA extraction, enabling high-quality outputs essential for more complex applications, such as whole-genome sequencing. This adaptability allows the HTGR to serve a wide range of molecular research needs, supporting both small and large-scale studies with a focus on quality and efficiency.

## 4 Methods

### 4.1 Strains and culture conditions

The *Aspergillus niger* wild-type strain ATCC 11414, obtained from the American Type Culture Collection (Rockville, MD), was cultivated on complete medium (CM) plates at 30°C for culture maintenance and spore preparation. Cultures were incubated on CM agar plates at 30°C for 4 days, and spores were harvested by washing with sterile 0.4% Tween 80. The *albA* and *oahA* mutants were generated through CRISPR genome editing as previously described (Yuan et al., 2024b).

### 4.2 1-well plate spore squashing

The 1-well plate spore squashing process begins by adding a small volume (typically 5 µL) of *Aspergillus niger* spores into the well. The Shear Applicator, precisely designed to fit the well, is aligned above the spores. One hand holds the plate steady on the bench, while the other hand presses and slides the Shear Applicator side to side to compress and break open the spores for 1 min. After squashing, the released genomic material is collected for downstream applications, such as microscopy and PCR testing.

### 4.3 96-well plate manual squashing

After pipetting 5 µL of *Aspergillus niger* spores into each well, one hand is used to hold the plate steady on the bench while the other hand carefully aligns the Shear Applicator with each well. The applicator is then pressed and slide horizontally across the plate for 1–2 min to compress and break open the spores in each well. Squashed spores can be directly transferred to a microscope slide for microscopy. For PCR testing, 40 µL of 1 × PBS with 0.05% Tween 20 is used to dilute and collect the extracted material.

### 4.4 Microscopy of squashed sample

The squashed spore sample, approximately 2–5 μL, is transferred from the well onto a microscope slide. A coverslip is gently placed over the drop to flatten the sample. The slide is then observed under an Olympus IX73 microscope to check for spore breakage and internal contents, with images captured at ×20 magnification.

### 4.5 PCR genotyping

PCR reaction was carried out using GoTaq Green Master Mix (Promega). For each reaction, 1 μL of squashed spore solution was used as the template in a 20 μL total reaction volume. The PCR conditions were an initial denaturation at 95°C for 2 min, followed by 35 cycles of 95°C for 30 s (denaturation), 55°C for 30 s (annealing), and 72°C for 1 min (extension), with a final elongation at 72°C for 5 min. Primers 11_F (5’-GAC​CAA​TGA​CAA​GAC​TCT​GTG​GGT-3’) and 12_R (5’-TCT​TCT​TCC​CCT​CCG​CAG​TGA​C-3’) were used for the *albA* gene, while primers 123_F (5’-CTT​CTG​GCC​CTT​CCT​TTC​TA-3’) and 124_R (5′-CAT​ACC​ATG​TAC​ATG​CCC​TT-3’) targeted the *oahA* gene.

### 4.6 HTGR controller configuration

The main parts of the HTGR controller include the stepper motor, motor driver, linear actuator, and load cell sensors. Specifications for each part, except for the motor driver, are summarized in Table 1. For the motor driver, any model compatible with NEMA 23 that operates within a current range of 1.0–4.2 A and a voltage range of 20–50 VDC is suitable. In our application, we used a resolution setting of 6,400 pulses per revolution. It is important to note that small PCB-type drivers are not recommended, as they do not dissipate heat effectively, even when equipped with a heat sink. In our case, the stepper motor required approximately 2 A at 24 V. As a power source, we used two standard power adapters, each dedicated to one stepper motor driver. The stepper motor drivers were connected to the stepper motors, and the control pins were linked to the MCU board to manage motor direction and pulses. When programming the MCU, it is crucial to define each pin number correctly according to the wiring. Each stepper motor was paired with a linear actuator. We used actuators with two different stroke lengths. For vertical movement, we used a 100-mm stroke actuator, which was sufficient to reach the 96-well plate. For horizontal movement, we used a 200-mm stroke actuator to provide enough space for changing the 96-well plate. To facilitate this, the Shear Applicator had to move to the side, allowing the user to easily replace the 96-well plate. Additionally, four load cell sensors were used, one placed at each corner of the bottom holder plate. The bottom holder plate was positioned below the 96-well plate. It is recommended to calibrate the load cell sensors using a known weight before installation. In our experiment, we used the load cell sensors to measure the force required to break spore cells during the initial stage. After this stage, we removed the sensors to maintain a balanced, flat bottom surface continuously.

**TABLE 1:** HTGR Hardware specification.

Load cell sensor		Stepper motor		Linear actuator A, B	
Capacity	200 N	Frame	NEMA 23	Frame	NEMA 23
Linearity	±0.25%	Max. Holding torque	1.2 N m	Max. Motor speed	1,500 rpm
Hysteresis	±0.25%	Max. Speed	1,000 rpm	Max. Motor torque	2.0 N m
Output	2 mV/V nominal	Max. Current per phase	2.8 A	Max. Speed	50 mm/s
Zero balance	±2%	Full step increment	1.8°	Travel distance per turn	2 mm
Ultimate overload	300% of capacity	Polarity	Bipolar	Stroke length	100 mm(A) 200 mm(B)
Weight	<14 g	Weight	680 g	Static load capacity	285.7 kg

### 4.7 HTGR controller SW usage

**FIGURE 9:**
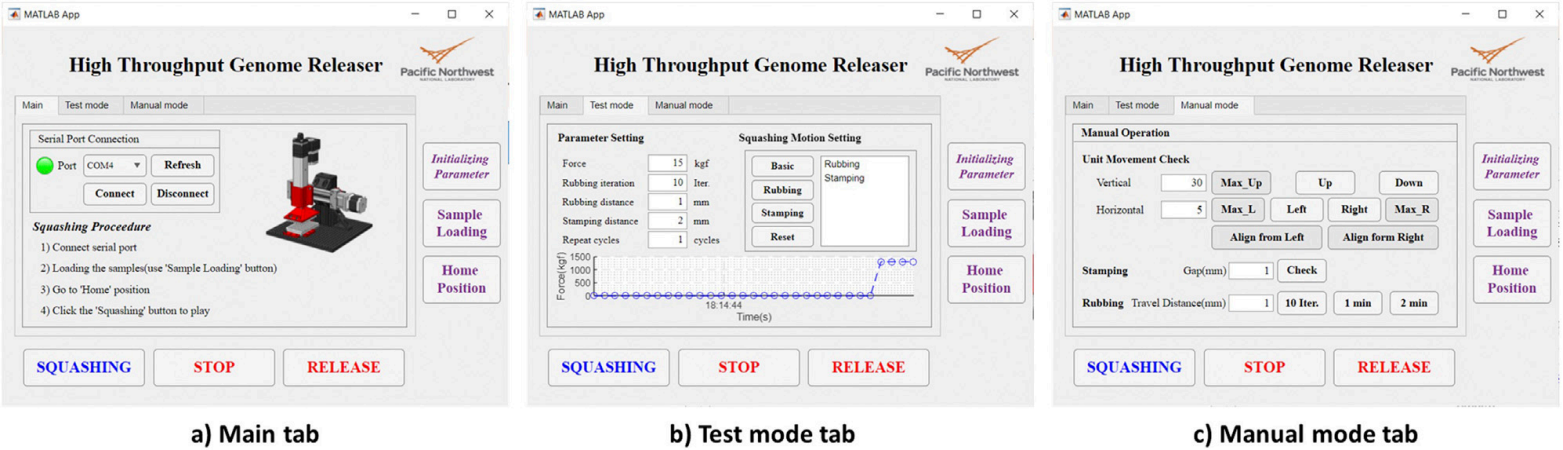
HTGR graphical user interface. **(A)** Main tab. **(B)** Test mode tab. **(C)** Manual mode tab.

To use the HTGR controller, the user must select several parameters related to communication and the squashing motion. The flowchart of the control software is shown in Figure 8B. For the communication settings, since the MATLAB GUI and Arduino communicate through a serial port, the user must select the appropriate port (Figure 9). Afterward, the user can program the squashing motion to fit their requirements or use the default settings. The user then sets parameters for the vertical (Y-axis) and horizontal (X-axis) movements that correlate with the squashing motion. Detailed explanation of these parameters described in Figure 8B and Table 2.

**TABLE 2:** HTGR Software parameters.

Parameter	Explanation (unit)	Related movement
f	Force (kgf)	Vertical (Y-axis) movement
N	Number of rubbing iterations	Horizontal (X-axis) movement
D	Rubbing distance (mm)	Horizontal (X-axis) movement
h	Stamping distance (mm)	Vertical (Y-axis) movement
C	Total repeat cycle	Both axis

Under the current hardware (stepper motor) and software setup, the frequency of the Shear Applicator movement during the rubbing motion follows the equation below.

$$f=\frac{k}{D\times T}$$

In the equation, f represents the rubbing frequency (Hz) of the Shear Applicator, D (mm) is the value of Rubbing Distance parameter, T (µs) is the stepper motor delay between steps, and k (nm) is an empirical constant, which is 55.84 in this setting. It was derived from the stepper motor configuration, accounting for micro-steps and movement per iteration. In this experiment, f was approximately 1.86 Hz.

The spore disruption process is straightforward. When the user initiates this process in the MATLAB app, it transfers the preset parameters to the MCU (Arduino, 2024), which then controls the stepper motors accordingly. First, the vertical stepper motor lowers the Shear Applicator to apply force to the 96-well plate until the desired force, as set by the user, is reached. Once the desired force is achieved, the programmed motion begins, squashing until it completes the specified number of cycles.

The graphical user interface of the MATLAB app consists of three tabs: Main, Test Mode, and Manual Mode (Figure 9). The HTGR software is designed to be easy to use. To operate it, the user needs to follow four steps.

1. Connect to the appropriate serial port.
2. Place the 96-well plate in the designated slot on the optical table.
3. Set the parameters for the desired squashing motion.
4. Click the “Squashing” button to start the process.

When the user clicks the ‘Initialize Parameters’ button, initial values for the squashing motion are loaded. The parameters used in this system are ‘Force,’ ‘Rubbing Iteration,’ ‘Rubbing Distance,’ ‘Stamping Distance,’ ‘Repeat Cycles,’ and ‘Programmed Squashing Motion.’ Each parameter is explained in Table 3. These parameters can be adjusted in the ‘Test Mode’ tab. When the squashing conditions change, such as when testing a new type of sample, the user can modify these parameters to achieve better results. In the ‘Test Mode’ tab, the user can adjust the squashing motion by clicking the motion buttons or selecting the default programmed motion. In the ‘Manual’ tab, users can test vertical and horizontal movements over specific distances and perform designed motions such as stamping and rubbing. All user input parameters are restricted by the software settings. The range for each parameter is summarized in Table 3. Users can easily adjust these settings within the software.

**TABLE 3:** Explanation of user defined parameters.

User defined parameters	Explanation	Input range
Force	Amount of force the user wants to apply to samples (96-well plate)	1–35 kgf (343.23 N)
Rubbing Iteration	The number of iterations during the rubbing process	1–600
Rubbing Distance	One-side travel distance of the Shear Applicator in the horizontal direction from the center point during the rubbing process	1–5 mm
Stamping Distance	One-side travel distance of the Shear Applicator in the vertical direction during the stamping process	1–10 mm
Repeat Cycles	The number of cycles the user wants to repeat the whole process	1–20
Programmed Squashing Motion	Combinations of motions the user programmed for the squashing process	N/A

### 4.8 1-well plate 3D printing using FDM and SLA

To create the 1-well plates required for spore squashing, we used two 3D printing methods: Fused Deposition Modeling (FDM) using a Prusa MK4 printer with PLA material, and SLA using a Formlab’s Form3 printer with Durable and Biomed Clear resins. The design process began in SolidWorks, where we finalized the geometry of the 1-well plate. Once the CAD model was complete, it was exported in STL format, a standard file type compatible with most 3D printing slicer software.

For the FDM process, the STL file was loaded into PrusaSlicer. We set a layer height of 0.1 mm, balancing surface quality and print time. A 100% infill density was specified to give the plate the rigidity needed to withstand the squashing forces applied during DNA extraction. PLA filament was chosen for these initial prototypes due to its availability, ease of use, and efficiency in testing different shapes for the well and applicator configurations. This initial FDM prototyping phase allowed us to assess the practical fit and mechanical interactions between the well and applicator, quickly identifying the most effective design features for consistent sample processing.

Following the shape exploration with FDM, we transitioned to SLA printing on a Formlabs printer to achieve higher resolution and test materials better suited for final applications. The STL file was imported into PreForm, where we adjusted the layer height to a finer 0.05 mm, further enhancing surface quality and ensuring a smooth, uniform well interior for optimal spore squashing. As in the FDM process, a 100% infill was chosen to maximize strength and prevent internal cavities that could compromise the well’s mechanical integrity during squashing.

### 4.9 1-well and 96-well plate CNC machining

Following initial evaluations, the 3D-printed 1-well plates did not meet the precision and durability required for repeated DNA extraction applications. Consequently, CNC machining was chosen to achieve the higher accuracy and reliability needed for the final components.

The CNC-machined single-well plates were fabricated in various configurations including cylindrical, rectangular, and oval, to match the specific requirements of different sample types. These parts were manufactured from PC, ABS, and PP, each selected for their mechanical properties suited to our experimental conditions. Tight tolerances of ±0.15 mm were maintained, with a surface roughness of 0.8 µm Ra across all components, ensuring consistent mechanical performance and uniform force distribution on the well surface during squashing.

To further enhance each material’s specific properties, vapor polishing was applied to polycarbonate parts, improving both transparency and surface smoothness. ABS components underwent bead-blasting, producing a smooth and flat finish critical for effective contact with the squashing applicator. In contrast, polypropylene parts received a standard finish, ensuring chemical resistance and durability for use with fungal spores.

Building on the success of the CNC-machined 1-well plates, a final 96-well plate was also manufactured from ABS to support high-throughput applications. ABS was selected due to its balanced mechanical properties, such as impact resistance, thermal stability, and a high-quality surface finish, which are essential for the repetitive stresses of the squashing process. The 96-well plate was CNC-machined to ensure precise alignment and consistent well dimensions, with each well maintaining the same surface roughness of 0.8 µm Ra as the single-well plates. This consistency across wells reduces variability and supports reliable DNA extraction across all 96 samples, making it suitable for large-scale genetic screening applications.

### 4.10 Mechanical design and implementation of high-throughput genome releaser system

The HTGR system’s mechanical assembly was designed in SolidWorks to ensure stability and precision for DNA extraction through controlled squashing and shearing motions. All main structural components, including the T-slotted aluminum frame, gusset brackets, linear stage, linear actuator, fixture plate, and fasteners, were sourced from McMaster-Carr. The frame consists of a vertical rail (38 × 76 × 152 mm), which is mounted to a 305 × 305 × 12.7 mm fixture plate with two black open gusset brackets (76 × 38 mm) on either side for stable support. Attached to the vertical rail is a horizontal rail (38 × 38 × 305 mm), connected with two additional gusset brackets. This horizontal rail holds the linear stage and the mounted linear actuator, with the Shear Applicator assembly bolted to the actuator rod. Together, these gusset brackets reinforce key connections, preventing misalignment under load and ensuring consistent force application across the wells. The red components, including the Shear Applicator mount and clamps, were 3D printed in PLA using a Prusa MK4 printer. The Shear Applicator itself is secured with M3 bolts and wing nuts, allowing for straightforward assembly and maintenance. Finally, the 96-well plate is clamped to the fixture plate using 1/4″-20 socket head bolts that pass-through 3D-printed brackets, providing a stable, flat surface for even force distribution during the squashing process.

## Data Availability

The original contributions presented in the study are included in the article/supplementary material, further inquiries can be directed to the corresponding authors.
